# Rhythm of the streets: a street classification framework based on street activity patterns

**DOI:** 10.1140/epjds/s13688-022-00355-5

**Published:** 2022-07-28

**Authors:** Tianyu Su, Maoran Sun, Zhuangyuan Fan, Ariel Noyman, Alex Pentland, Esteban Moro

**Affiliations:** 1 Department of Urban Studies and Planning, Massachusetts Institute of Technology, Cambridge, MA USA; 2 Graduate School of Design, Harvard University, Cambridge, MA USA; 3 Senseable City Lab, Massachusetts Institute of Technology, Cambridge, MA USA; 4 Media Laboratory, Massachusetts Institute of Technology, Cambridge, MA USA; 5 Department of Mathematics and GISC, Universidad Carlos III de Madrid, Madrid, Leganés, Spain; 6 Department of Geography, University of Hong Kong, Hong Kong, China

**Keywords:** Street activity, Temporal patterns, Street classification, Mobile phone GPS data, Clustering, FCM, Urban management

## Abstract

As the living tissue connecting urban places, streets play significant roles in driving city development, providing essential access, and promoting human interactions. Understanding street activities and how these activities vary across different streets is critical for designing both efficient and livable streets. However, current street classification frameworks primarily focus on either streets’ functions in transportation networks or their adjacent land uses rather than actual activity patterns, resulting in coarse classifications. This research proposes an activity-based street classification framework to categorize street segments based on their temporal street activity patterns, which is derived from high-resolution de-identified and privacy-enhanced mobility data. We then apply the proposed framework to 18,023 street segments in the City of Boston and reveal 10 distinct activity-based street types (ASTs). These ASTs highlight dynamic street activities on streets, which complements existing street classification frameworks, which focus on the static or transportation characteristics of the street segments. Our results show that a street classification framework based on temporal street activity patterns can identify street categories at a finer granularity than current methods, which can offer useful implications for state-of-the-art urban management and planning. In particular, we find that our classification distinguishes better those streets where crime is more prevalent than current functional or contextual classifications of streets.

## Introduction

While streets bear a critical role of ensuring a functionally connected and accessible city, they also accommodate the livelihood and experience of cities, as emphasized by Lynch [1], Gehl and other scholars [2–9]. In recent years, North American cities, such as Boston, Philadelphia, and Chicago, have seen the emergence of the “Complete Streets” concept, where streets act as “both great places to live and sustainable transportation networks” [10, 11].

Many cities have adopted different street classification frameworks to capture street networks’ functions and characteristics. These frameworks guide the allocation of transportation resources, regional planning, and design of livable streetscapes [11]. Table 1 exemplifies a few typical street classification frameworks [11]. In general, there exist two main classification frameworks: functional frameworks and contextual frameworks. While functional frameworks capture the transportation aspects of streets, contextual frameworks aim to capture public life by considering streets’ spatial contexts. As an example of functional frameworks, the Census Feature Class Codes (CFCC) system classifies street segments into seven main categories based on their transportation functions. On the other hand, the contextual classification framework proposed in Boston Complete Streets: Design Guidelines [10] categorizes streets into nine types based on their geographical locations and adjacent land uses.

**Table 1:** Functional and contextual street classification frameworks

Framework	Organization	Document/System	Street types	Transportation aspect	Public life aspect
Functional	U.S. Census Bureau	Census Feature Class Codes (CFCC)	– Primary Highway with Limited Access	Yes	No
			– Primary Road without Limited Access		
			– Secondary and Connecting Road		
			– Local, Neighborhood, and Rural Road		
			– Vehicular Trail		
			– Road with Special Characteristics		
			– Road as Other Thoroughfare		
Functional	Federal Highway Administration (FHWA)	Highway Functional Classification Concepts, Criteria and Procedures (2013)	– Arterial	Yes	No
			– Collector		
			– Local street		
Contextual	City of Boston	Boston Complete Streets: Design Guidelines (2013)	– Downtown Commercial	Yes	Yes
			– Downtown Mixed-Use		
			– Neighborhood Main Street		
			– Neighborhood Connector		
			– Neighborhood Residential		
			– Industrial		
			– Shared Streets		
			– Parkways		
			– Boulevards		
Contextual	City of Philadelphia	Philadelphia Complete Streets Design Handbook (2017)	– High-Volume Pedestrian	Yes	Yes
			– Civic/Ceremonial Street		
			– Walkable Commercial Corridor		
			– Urban Arterial		
			– Auto-Oriented Commercial/Industrial		
			– Park Road		
			– Scenic Drive		
			– City Neighborhood		
			– Low-Density Residential		
			– Shared Narrow		
			– Local		

The functional frameworks, which largely depends on transportation demand, does not provide enough insights for the future pedestrian friendly urban development. The contextual frameworks, mostly resorting to static characteristics, ignore the valuable micro-level urban dynamics [12]. The locations and land uses are not capable of expressing the temporal pattern of street activities. For example, a Boston Downtown street segment next to a large departmental store and another street segment next to a local grocery can show distinct street activity patterns, even though both are adjacent to commercial land uses. Besides, typical contextual frameworks use streets rather than streets segments as their classification unit, which bears a lower resolution than functional frameworks (e.g., CFCC). For longer streets that contain a lot of segments, this approach may overlook the spatial variations of street activities. Moreover, contextual frameworks’ classification rules vary significantly across cities (see Table 1), making it hard to apply them in different urban contexts.

Recently, advancements in urban sensing and location intelligence made it possible to establish fine-grained street classification frameworks highlighting dynamic street activity patterns. These patterns demonstrate how individuals use streets to live, play, eat, rest, shop, as well as conduct a wide range of other activities.

The activity-based street classification frameworks have their roots at the intersection of urban and social science studies. By observing urban life, Gehl in [2] demonstrates how street activities occur in outdoor spaces and how places’ physical characteristics (e.g., buildings, sidewalks, and street furniture) influence the way people behave. Similar approaches have been applied by Gehl and Svarre in [4] and Whyte in [6]. However, constrained by the tools and methods, these studies mostly focus on concentrated areas or a few streets, and do not easily scale.

In recent years, the increasing availability in location data enable researchers and practitioners to study human activities in larger urban scales that go beyond neighborhood. Earlier research focuses on a lower resolution using mainly Call Detail Records (CDR) data [13–15], while later studies use higher-resolution telecom data and GPS data [16–18]. While lower-resolution location data are capable of analyzing human activities in medium to large spatial units, such as urban grids or census tracts, higher-resolution data can support research at smaller spatial units, such as street segments or blocks. For example, Ratti et al. in [14] introduce the potential applications of location-based services (LBS) data in the urban studies field and investigate the temporal patterns of urban activities in Milan by observing CDR data. More recently, the authors in [19] studied the phenomenon of social segregation at individual site level using de-identified high-resolution GPS data.

Among the studies trying to understand urban dynamics using large-scale location data, many of them investigated latent patterns in cities [13–16, 20–26]. For example, Zhu et al. in [20] proposed using street segments as a linear unit to investigate small-scale urban dynamics, and clustered main street segments of Beijing into nine types using taxi pick-up and drop-off GPS data. D’Silva et al. [21] extracted the temporal activity patterns on both venue and ward scales from the Foursquare mobility data and used a k-nearest neighbor model to predict the performance of new business venues. Pei et al. [13] utilized the mobile phone calling patterns on the urban cell scale to classify land uses via a semisupervised fuzzy c-means (FCM) clustering approach.

**Figure 1:**
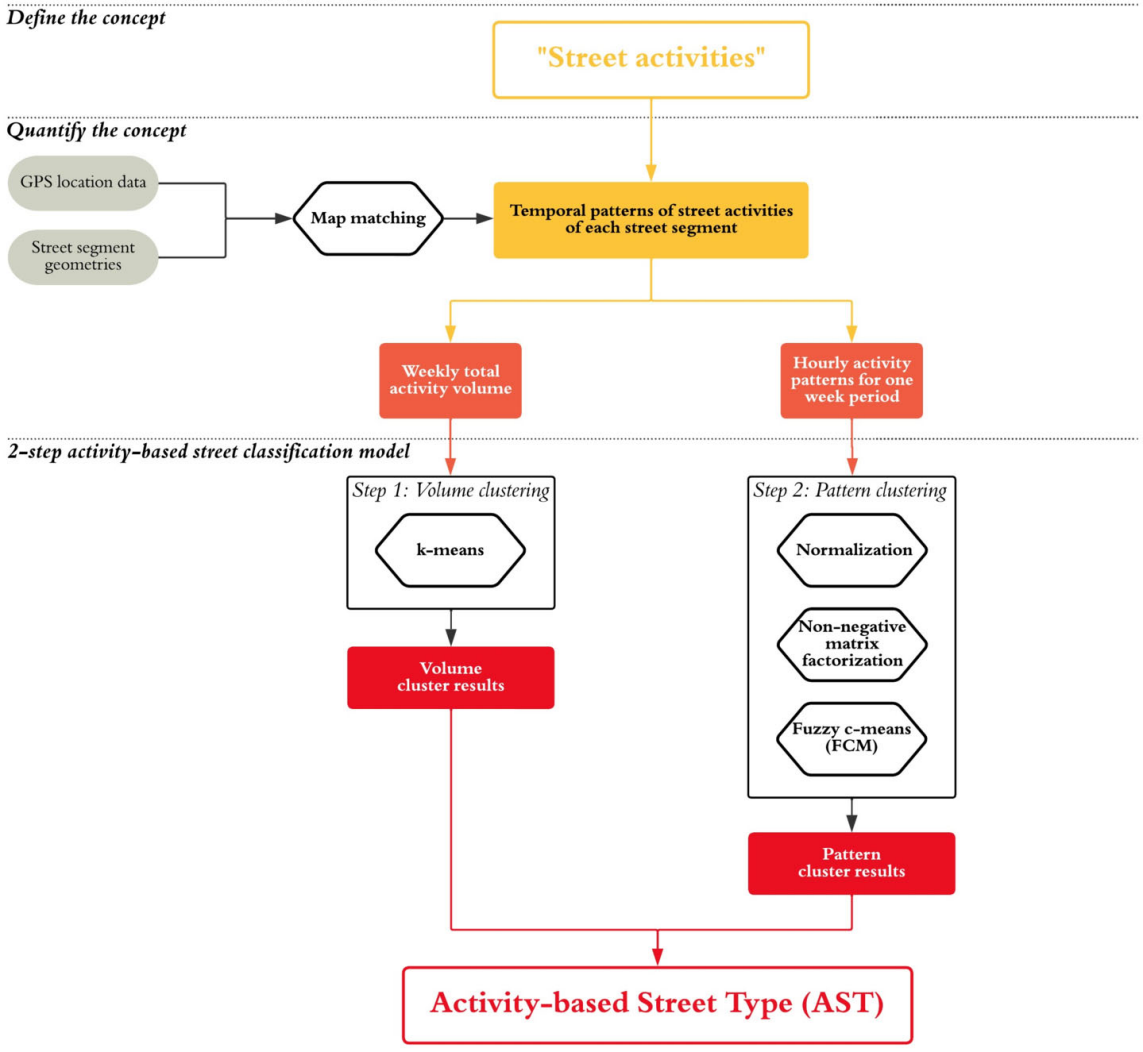
The framework of activity-based street classification

Building on existing street classification frameworks and the recent progress in finer-grained urban mobility data, this research proposes a scalable and reusable street classification framework based on the temporal patterns of street activities on street segments. Figure 1 portrays the proposed framework. First, we define “street activities” as non-motorized human activities that take place on streets or in adjacent public spaces and buildings. Second, we quantify the “rhythms of streets,” the temporal patterns of street activities, using anonymized and aggregated GPS mobility data. Third, we propose a two-step activity-based classification model to classify 18,023 street segments in Boston into distinct activity-based street types (ASTs). We also discuss how the proposed framework can complement current functional and contextual street classification frameworks.

## Data description

### Street segments of Boston

We use street segments as the basic study unit to address the finer-grained spatial variations along streets. A total of 19,212 street segments of Boston are downloaded from Analyze Boston,^1^ the City of Boston’s open data hub. As this study focuses on *non-motorized street* activities, we exclude 1189 segments that are only for vehicle traffic uses. This selection is based on the CFCC of each street segment.

Detailed criteria are available in Appendix Table 6.

### Street activities

The GPS location data comes from anonymized users who have opted-in to provide access to their LBS data through a GDPR-compliant framework. Data was shared in 2017 under a strict contract with Cuebiq through their Data for Good program where they provide access to de-identified and privacy-enhanced mobility data for academic research and humanitarian initiatives only.

Both geo-tagged social media data and GPS location data have been widely used for detecting latent patterns in cities. Social media data has been widely used to describe activities associated with specific places such as parks, restaurants, coffee places, and transit stations [21, 24, 25, 27–29]. In comparison, GPS data provides more flexibility to be aggregated at different spatial unit across spatial scales [19]. Admittedly, both data are biased towards certain user groups, given that GPS data has a much larger sample size and wider time range, we are able to present the street activities that cover more than certain venues that have social media presence, and beyond their scheduled operation hour.

The data we use covers 12 weeks from October 2, 2017 to December 24, 2017. It is processed to get the stays (stops) of individuals so it contains an inferred device location (i.e., latitude and longitude), a timestamp of the starting time $t_{i}$, and a dwell time $d_{i}$ (see [19] for more details about the data).

As briefly discussed in Sect. 1, street activities are defined as the non-motorized human activities that happen on streets or at places accessible from streets in this research. We choose $d_{i}$, the dwelling time, as the key indicator to filter the raw data and focus on the street activities. We use the 5 to 120 minutes as a threshold to exclude invalid activities such as driving through a street or staying at home or work for a long time. A total of 86,220 unique users and 1,419,079 stays are selected based on these criteria.

The filtered GPS stays are then mapped to their nearest street segments via a matching method [20, 30]. We set the threshold of matching to be 50 m since we focus on the activities happening on street segments and their adjacent spaces. This process keeps 82,620 unique users and 1,333,390 stays. Through this process, the point-based stays are linked to street segments. The processed data is then used to represent spatial-temporal patterns of street activities.

### Point of interest (POI) data

The point of interest (POI) data used in this research is a combination of two major sources: Reference USA and OpenStreetMaps. Reference USA provides a comprehensive list of registered businesses located in Boston while contains very little information about public amenities and outdoor places (e.g., parks). To address this limitation, locations of public amenities (i.e., kindergarten, schools, colleges, and universities) and outdoor open spaces (i.e., parks, gardens, and camping sites) are extracted from OpenStreetMaps. We then use these POI locations as a complement of the business POI data from Reference USA and compile a complete POI dataset.

## Methods

### Representation of street activity rhythms

Central to the activity-based classification framework is to represent the spatial-temporal patterns of street activities for each street segment. Each street segment’s activities are represented as a time-ordered sequence of 168 hours in a week (i.e., from Monday to Sunday). Following previous literature [20, 21], We represent the street activity in 1-hour granularity. We coded matched street activities into the hour or hours it happened based on its timestamp and dwelling time, resulting in $R[j,\tau ]$, which denotes the total street activity amount along the segment *j* during hour *τ*. To incorporate the underlying impacts of occasional fluctuations and construct a more robust longitudinal representation, the 168-hour activity sequence $A[j,\tau ]$ of street segment *j* on hour *τ* is calculated using multi-week activity records. Equation (1) shows the calculating procedure.

1 $$ A[j,\tau ]=\frac{\sum_{k=1}^{K}R[j,\tau ]}{K}\quad (j=1,2,\ldots,J;\tau=0,1,\ldots,T-1;k=1,2, \ldots,K) , $$

where $A[j,\tau ]$ denotes the average hourly activity counts during hour *τ* on street segment *j*; $R[j,\tau ]$ is the raw hourly activity counts during hour *τ* for each week. In Eq. (1), *J* is the total number of street segments (in this case, $J = 18{,}023$); *T* is the total hours considered in the pattern representation (in this case, $T = 168$); and *K* is the number of weeks aggregated in our research (in this case, $K = 12$).

Then, based on the average hourly activity counts generated with Eq. (1), we also construct a volume vector to represent the average weekly total activity volume of street segment *j*, given by Eq. (2):

2 $$ V[j]=\log_{10}\Biggl(1+\sum_{t=0}^{T-1}A[j, \tau]\Biggr)\quad(j=1,2,\ldots,J;\tau=0,1,\ldots,T-1) , $$

where $A[j,\tau ]$ is the average hourly activity counts during hour *τ* on street segment *j*. We logarithm transform the volume to incorporate the large variations.

### Activity-based street classification framework

Building on early efforts towards understanding urban dynamics, we propose an activity-based framework to classify street segments. It is based on segments’ street activity rhythms, namely the average weekly total activity volume $V [j]$ and the average hourly activity sequence $A[j,\tau ]$.

#### Activity volume clustering

We first apply an FCM clustering procedure to the volume vector $V [j]$, in order to classify the segments into $C_{1}$ volume clusters. In this process, the elbow method is employed to determine the optimal value of $C_{1}$, which tests different cluster sizes $C_{1}$ and compares the total within-cluster variation.

#### Activity pattern clustering

As the volume clustering process captures the variations of activity volumes among street segments, we first normalize $A[j,t]$, the activity sequence, in order to focus on the pattern dissimilarity of street activities. We then apply a non-negative matrix factorization (NMF) approach to reduce the dimension of the normalized 168-dimension activity sequence to make the following clustering process less computationally costly. Finally, an FCM clustering process is applied to identify $C_{2}$ pattern clusters. We combine the elbow method and the Silhouette method [31] to determine the optimal $C_{2}$ value.

Once we obtain the optimal $C_{1}$ and $C_{2}$ values for volume clusters and pattern clusters, we map them and generate a raw matrix of street types, including $C_{1} \times C_{2}$ final ASTs.

## Results

In this study we present and test a new street classification framework to explore the underlying rhythms of urban public life. This section elaborates the application of the proposed framework on the case study of Boston.

### Activity volume clustering results

We use the volume vector $V [j]$ to represent the weekly total activity volume of street segment *j* in Boston. Through exploratory data analysis, we find that a proportion of street segments have very low street activity intensity. In order to improve the model robustness, we group them as the *Subdued* type. Then, the FCM clustering approach is employed to identify the volume clusters excluding the *Subdued* segments. The elbow method helps us choose 3 as the optimal $C_{1}$ value (see Appendix Fig. 12).

**Figure 2:**
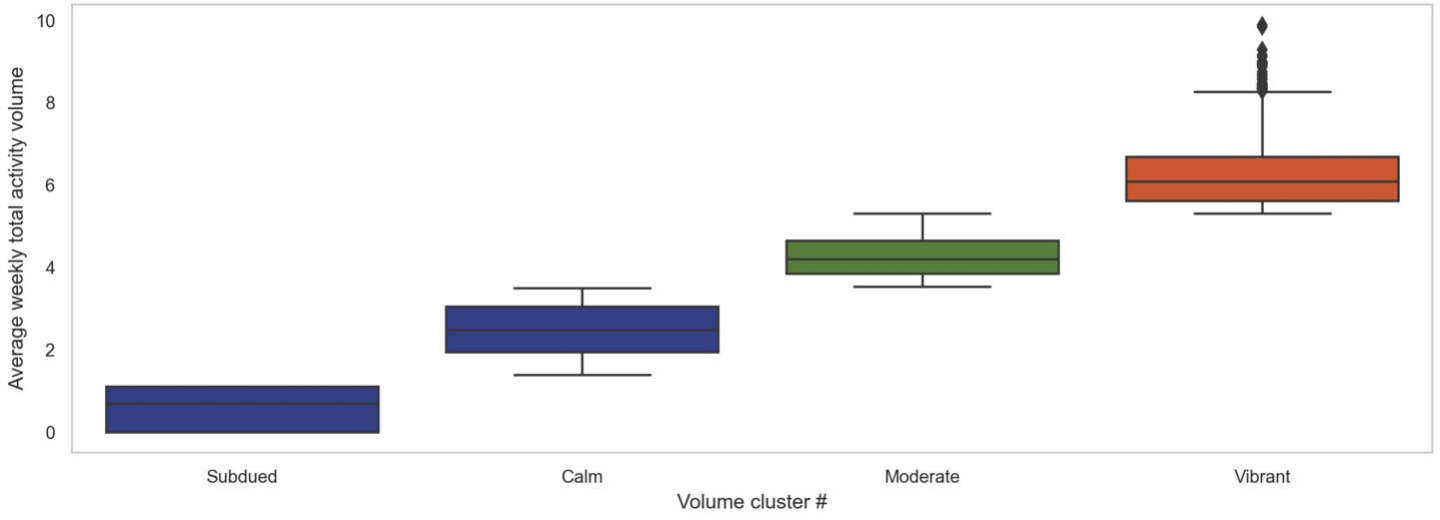
Identification of volume clusters. Boxplots indicating the distribution of average weekly total activity volume in the identified four Volume Clusters, where *Subdued*, *Calm*, *Moderate* and *Vibrant* Clusters are drawn from left to right

**Figure 3:**
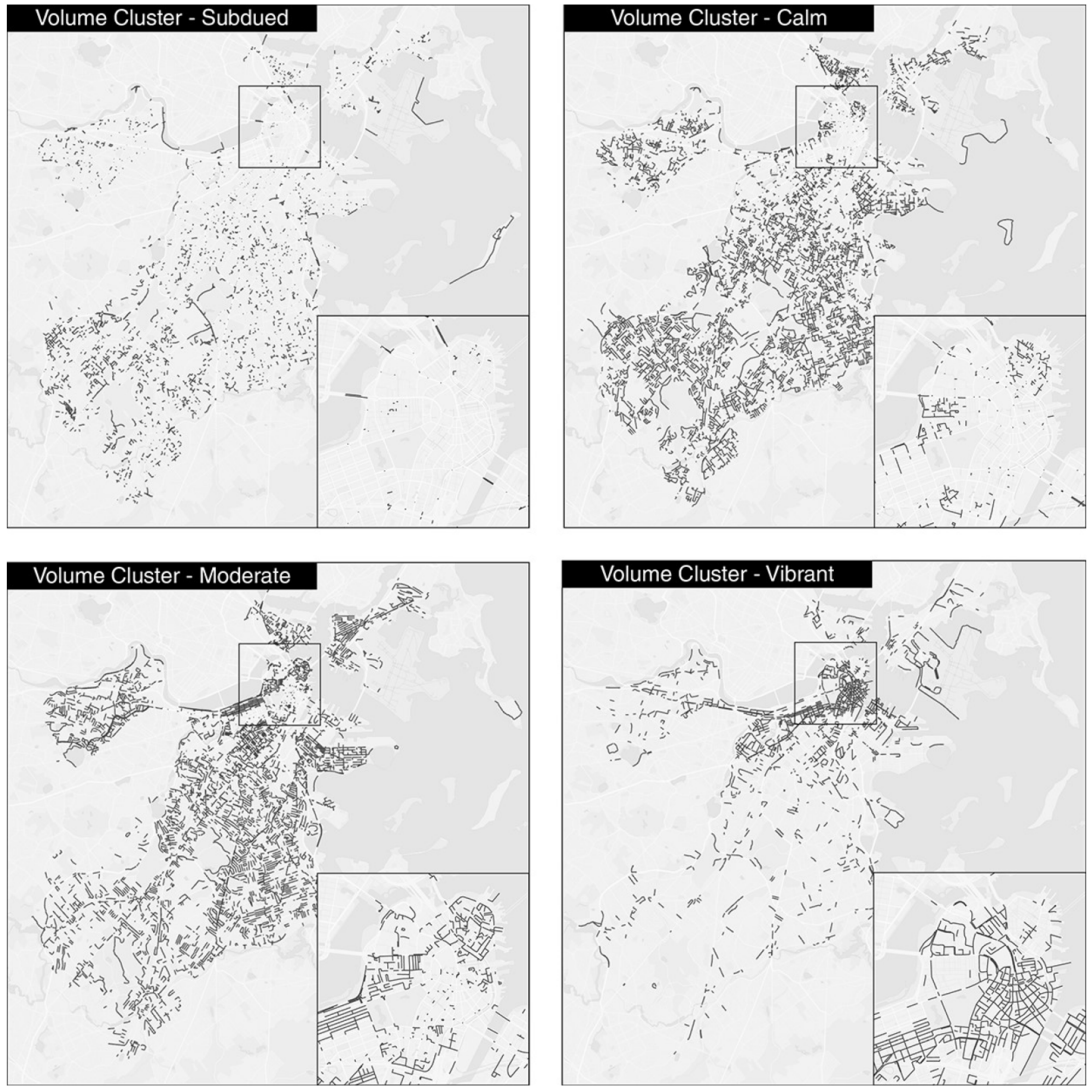
Spatial distribution of the volume clusters. The detailed map in each frame shows a zoom-in of Boston’s downtown area

Figure 2 indicates the identified activity volume clusters, and Fig. 3 shows their spatial distribution. *Subdued* segments are mostly small alleys, tunnels and local streets in suburban areas. The *Calm* type contains the low-volume street segments, which are mainly located within residential areas. For the *Moderate* type, most segments are on main streets in residential areas or on important connecting roads. Lastly, the intensively visited segments close to the downtown and medical districts belong to the *Vibrant* cluster.

### Activity pattern clustering results

We apply NMF to the normalized 168-dimension activity sequence and reduce it to 10 dimensions. We identify the optimal dimension candidates with Elbow method as 8, 10 and 12. Then clustering is performed for each candidate and examined by exploring if the spatial distribution of the clustering result makes sense. We classify street segments into $C_{2}$ groups based on their activity patterns. With the elbow method and the Silhouette methods together, we determine the optimal $C_{2}$ value as 3 (see Appendix Fig. 13).

**Figure 4:**
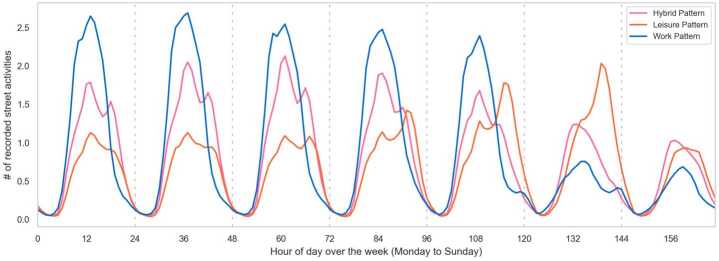
Identification of pattern clusters. The line graphs show the average street activity rhythms of *Work, Hybrid*, and *Leisure* pattern clusters

Figure 4 shows the average street activities of three pattern clusters. The *Work* pattern shows a single daily peak on weekdays and has apparently low values on the weekend. The *Hybrid* pattern has two distinguished peaks during weekdays and one peak during the weekend. Also, a *Leisure* pattern is characterized by increased activity volumes on Friday and Saturday with peak hours in the evening.

### Resulting ASTs

Each street segment, except the ones in the *Subdued* type, belongs to one of the volume clusters, denoted by $c_{1}$, and one of the pattern clusters, denoted by $c_{2}$. Pairing them together, we classify the segments into 10 distinct final ASTs (i.e., *Subdued*, *Hybrid-Calm*, *Leisure-Calm*, *Work-Calm*, *Hybrid-Moderate*, *Leisure-Moderate*, *Work-Moderate*, *Hybrid-Vibrant*, *Leisure-Vibrant*, and *Work-Vibrant*).

**Figure 5:**
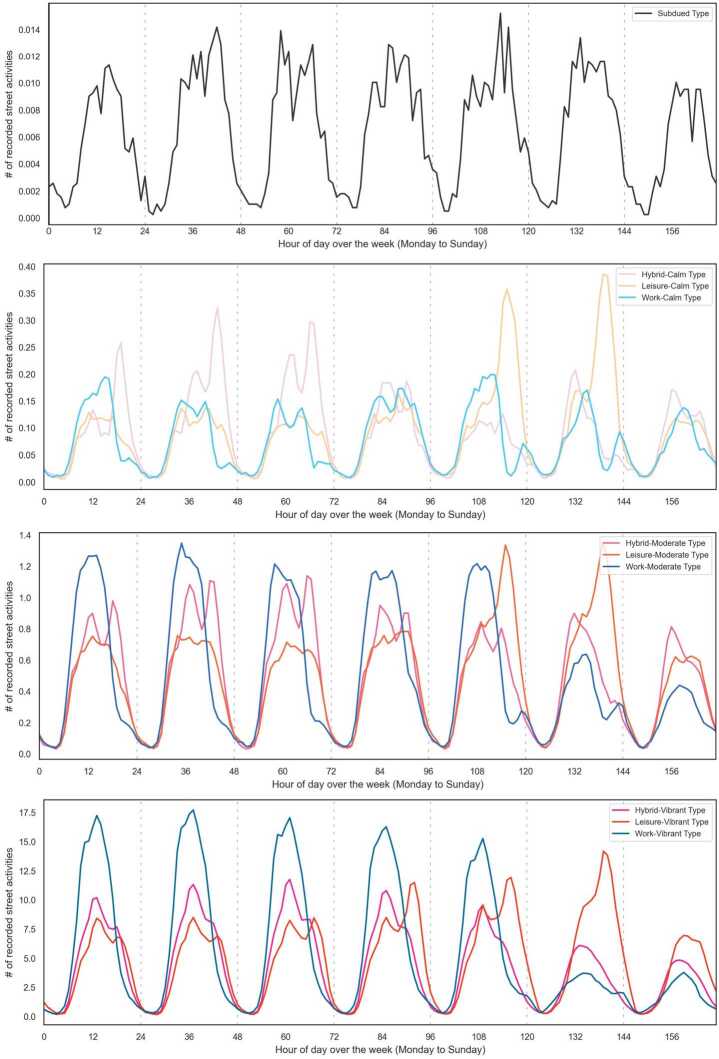
Average street activity rhythms of ASTs

**Figure 6:**
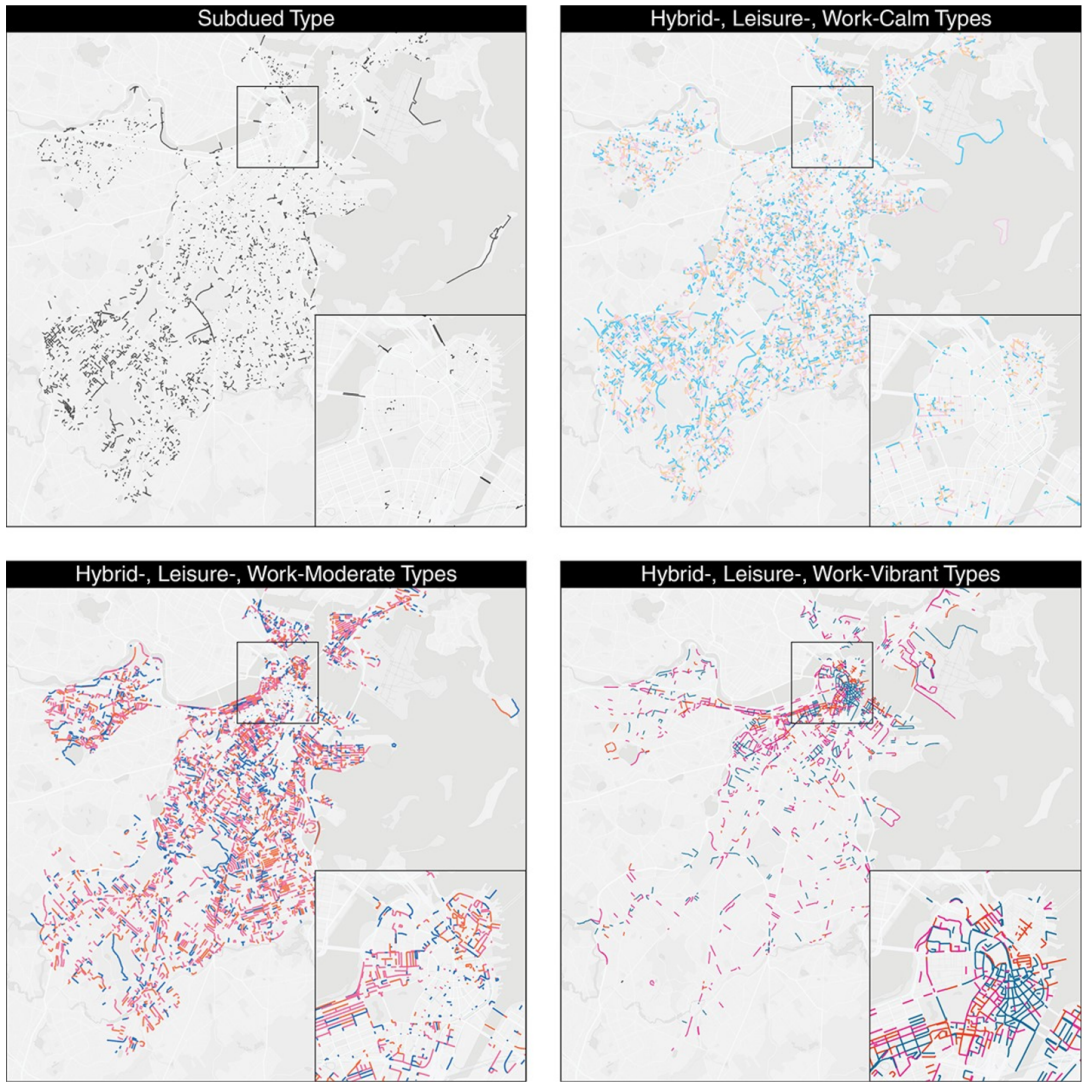
Spatial distribution of ASTs

Table 2 summarizes each AST’s size, street activity rhythms, characteristics, transportation functions (i.e., CFCC), and adjacent land uses. Figures 5 and 6 illustrate the average street activity rhythms and spatial distribution of ASTs, respectively.

**Table 2:** Description of resulting ASTs

AST	Pedestrian activity rhythms	AST size	Transportation function	Adjacent land use
Subdued	Low total visit volume and peak hours at noon everyday	3878	Similar to the overall distribution	Nearly 10% more residential and 10% less in commercial
Hybrid-Calm	Peak hours everyday around the noon time and evening with a drop in activity volumes on Fridays	2575	Similar to the overall distribution	Similar to the overall distribution
Leisure-Calm	Peak hours on Friday and Saturday evenings	1959	More Local, Neighborhood, and Rural Road	15% more residential and less commercial
Work-Calm	Peak hours in mornings and afternoons	2860	Slightly bigger proportion of Secondary and Connecting Road	More residential and less commercial
Hybrid-Moderate	Peak hours on weekday noon and evening and weekend noon	2211	Slightly bigger proportion of Local, Neighborhood, and Rural Road and less Secondary and Connecting Road	More commercial and institutional land use
Leisure-Moderate	Peak hours around Friday and Saturday evenings	1339	Slightly smaller proportion of Local, Neighborhood, and Rural Road and more Secondary and Connecting Road	Less residential and more commercial
Work-Moderate	Peak hours around noon with a marked drop on weekends	1487	Slightly smaller proportion of Local, Neighborhood, and Rural Road and more Secondary and Connecting Road	Much less residential and more commercial, institutional and industrial
Hybrid-Vibrant	Peak hours in noon with a drop on weekends	744	15% smaller proportion of Local, Neighborhood and Rural Road and 10% more Secondary and Connecting Road	35% less residential and much more commercial, institutional, and transportation
Leisure-Vibrant	Very high peaks showing at noon and evening	341	Much Smaller proportion of Local, Neighborhood, and Rural Road and more Secondary and Connecting Road	40% less residential and much higher proportion of transportation, institutional and industrial
Work-Vibrant	Peak hours in noons and a drop on weekends	629	Less Local, Neighborhood, and Rural Roads and more Secondary and Connecting Road	Much less residential and more commercial, institutional and transportation

### Case study

**Figure 7:**
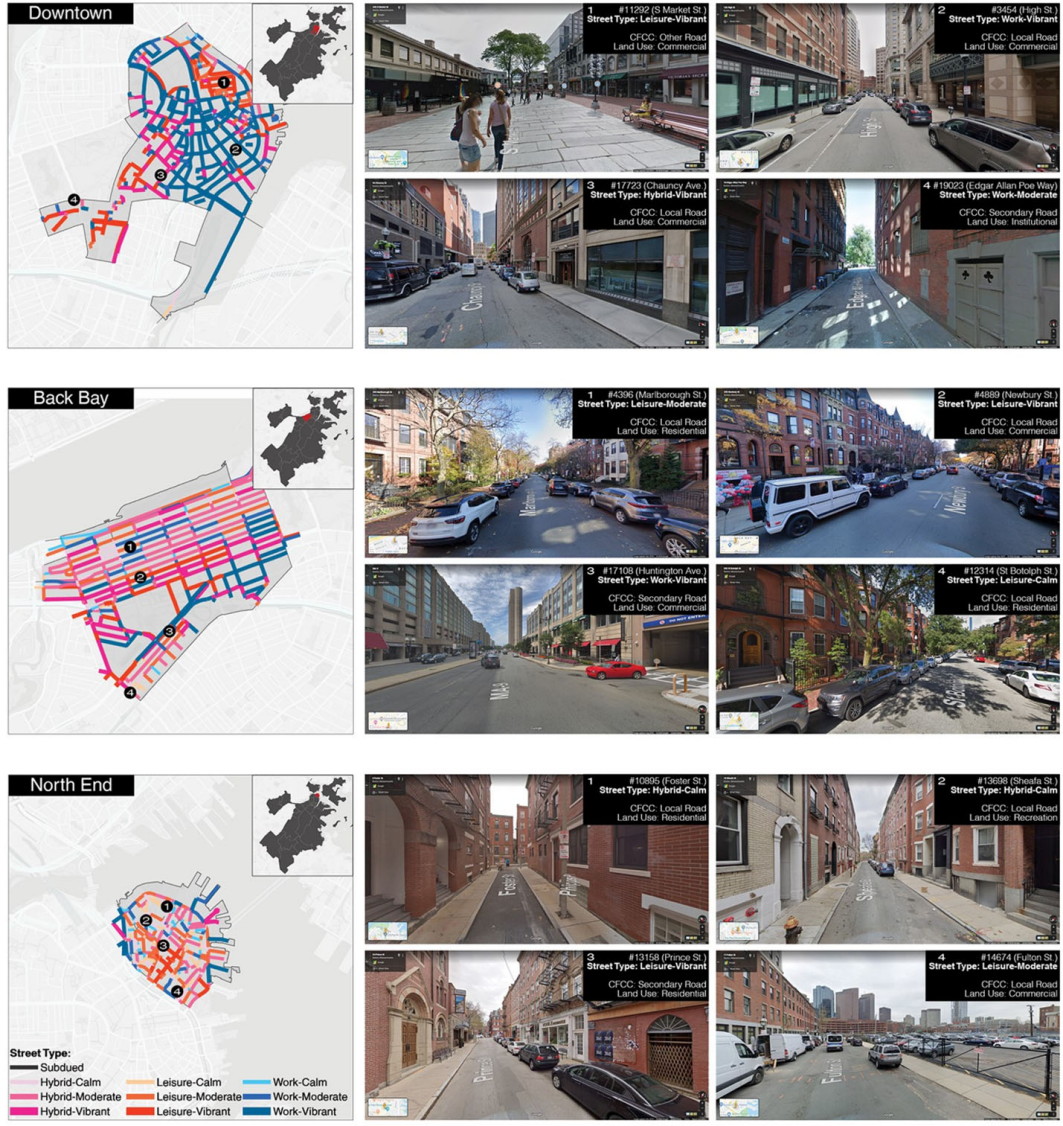
ASTs of three example Boston neighborhoods and information of selected street segments, including Google Street View Images, ASTs, street categories in the functional classification system (i.e., CFCC), and street categories in contextual classification framework (i.e., land used-based framework)

Figure 7 shows the ASTs of street segments in three selected Boston neighborhoods: Downtown, Back Bay, and North End.

The ASTs implies the following findings. First of all, ASTs show clear spatial heterogeneity at the local scale. Street segments that are close to each other can belong to different ASTs. This indicates diverse street activity rhythms within a small spatial limits. To quantify how street segments’ ASTs are correlated with their locations, we calculate Moran’s I indices for the volume and pattern clustering results, i.e., Volume Moran’s I and Pattern Moran’s I. Downtown’s street segments, marked by their substantial variations of activity volumes, have the smallest Volume Moran’s I value among the three neighborhoods. The small value (0.167) indicates the distribution of volume clusters are the least correlated with their locations. On the other hand, the clustered *Work* segments have the highest Pattern Moran’s I value (0.241) at Downtown Boston.

For the Back Bay area, the relatively regular distribution of activity volume contribute to the highest Volume Moran’s I (0.368). As one of the main retail clusters in Boston, roughly half of the street segments belong to the *Vibrant* types, which are mainly located in the middle of the neighborhood. In the northern part of Back Bay and on its southern boundary, many street segments in residential areas are classified into the *Calm* types. On the other hand, Back Bay’s limited Pattern Moran’s I value (0.107) reflects its heterogeneous activity pattern types. The North End ranks between these two neighborhoods for both Volume and Pattern Moran’s I values (0.329 and 0.123, respectively).

Zooming in to the selected street segments in each neighborhood, we find that the ASTs can help us infer the local functions and street activity types. Segment 3454, located inside the Boston’s Financial District within Downtown, is classified as a *Work-Vibrant* type. This type has high and stable activity volumes during working hours and relatively low volumes during other periods, including weekends. On the contrary, segment 11292 is located at the Quincy Market, a well-known shopping and tourism hot-spot in Boston. The high volume activities in afternoons and weekends result it in the *Leisure-Vibrant* type. This inferential capacity is especially helpful for visually similar street segments. For example, segment 10895, 13698, and 13158 are three visually very similar North End segments. However, they belong to two different Street Types. This result is reasonably caused by the physical and social functions of the streets.

In addition, ASTs reveal complementary information for functional and contextual frameworks. First, segments within the same functional and contextual categories can show distinct activity patterns. For example, segment 3454 and 17723 in Downtown Boston are in the same functional (i.e., Local Road) and contextual categories (i.e., Commercial). However, 3454 belongs to Work-Vibrant while 17723 is a Hybrid-Vibrant segment. This is possibly due to the stores and restaurants on 17223 which contributed certain street activity during the day.

Second, functionally or contextually distinct street segments can share similar street activity patterns and are classified into the same AST. For example, segment 11292 in Downtown and 4889 in Back Bay are in different functional street categories (i.e., Other Road for 11292 and Local Road for 4889), but both belong to the Leisure-Vibrant type in the AST framework. Both street segments feature highly vibrant commercial activity: 11292 is located in the Quincy Market and 4889 is a part of the Newbury Street. Their similar social function might explain why they are classified into the same AST. These results show that the proposed AST framework can capture information that is missing in existing street classification frameworks and add a layer of granularity to the understanding of pedestrians’ usage of streets.

## Discussion

### A comparison between ASTs and functional street categories

**Figure 8:**
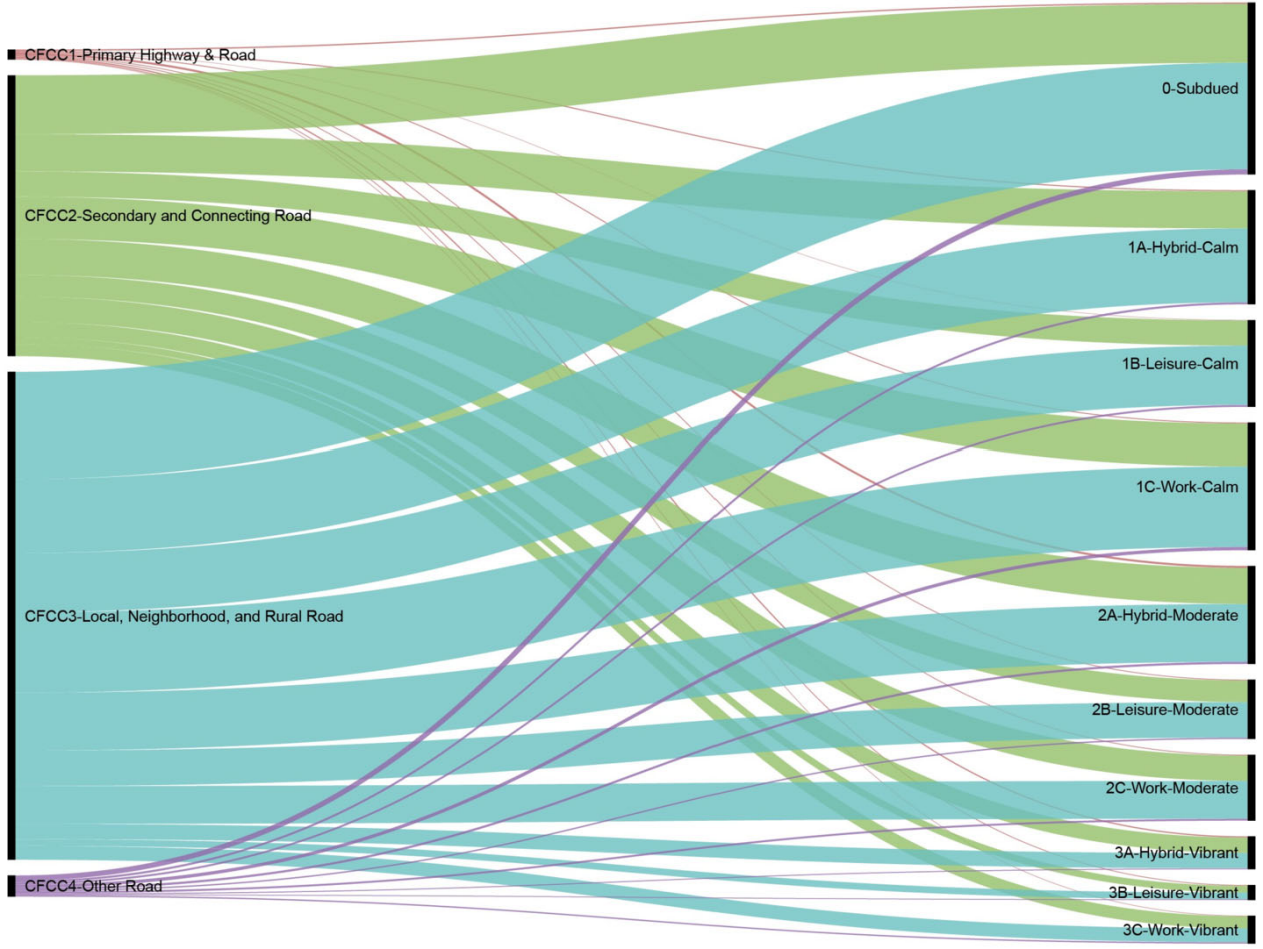
The comparison between functional street types (on the left side) and AST results (on the right side)

We compare the ASTs of Boston’s street segments with the aforementioned functional street categories indicated by CFCC. We adopt the normalized mutual information (NMI) [32] index, which evaluates information partitioning, to quantitatively compare street categories in these two classification frameworks. NMI score ranges from 0 to 1, where 0 represents partitions are completely different and 1 represents perfect overlap between partitions. The NMI score between CFCC and ASTs is 0.008, indicating little correlation between two categories. Figure 8 shows that the commonly used functional street categories do not provide adequate information for the AST results. As the largest functional category, “Local, Neighborhood, and Rural Road” contains more than half of the street segments, and they distribute roughly equally amongst the 10 ASTs. It reveals that, even though the functional street type denotes the street’s (or the street segment’s) role in urban transportation systems, it does not necessarily illustrate how individuals, especially pedestrians, use that street.

### A comparison between ASTs and contextual street categories

As one of the mainstream contextual classification systems, land use-based classification framework has been applied in many practices. For example, Boston employed the land use-based classification framework in designing its Complete Streets: Design Guidelines. In this section, we investigate the question of how much the adjacent land use of a street segment can explain the variation of its street activity rhythms.

**Figure 9:**
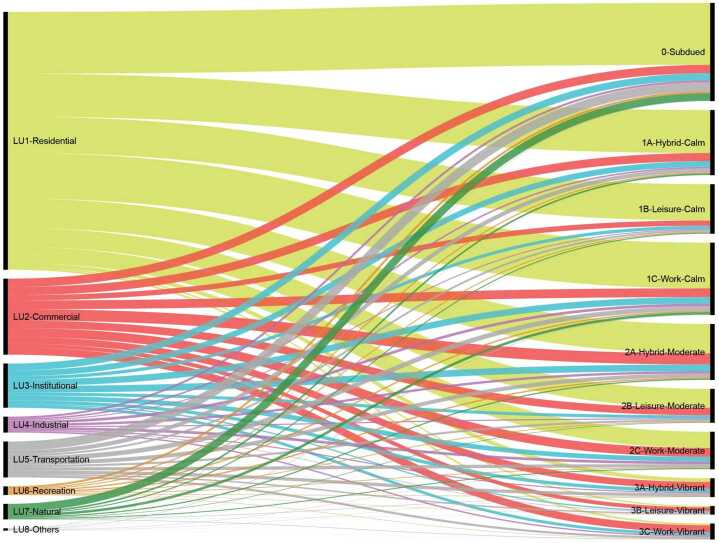
The comparison between land use-based street types (on the left side) and AST results (on the right side)

Boston outlines nine contextual street types (e.g., Downtown Commercial, Neighborhood Main Street, and Shared Streets) but provides only few examples associated with each street type. We then represent the contextual street category of each street segment by its adjacent land use (e.g., residential, commercial, and institutional), in order to have a larger sample to investigate. The NMI index between land use types and ASTs is 0.04, representing a slightly greater overlap. Figure 9 summarizes the comparison between segments’ contextual categories and their ASTs. As shown, land uses carry more information about streets’ social functions than functional street categories. For example, most of the “residential” street segments are classified into the *Calm* and *Moderate* types, which have relatively low and medium activity intensity. Moreover, roughly half of the “commercial” street segments belong to the Vibrant types. It is also worth mentioning that a large proportion of “natural” street segments are in the Subdued type, which shows a pattern with low activity volume, indicating that these street segments are less used by pedestrians than others.

Nevertheless, certain information regarding street activities can not be explained by adjacent land uses. Although many “commercial” street segments belong to the *Vibrant* ASTs, another half of these segments are classified into the *Calm* and *Moderate* ASTs. It entails that spatial proximity to commercial land-use cannot guarantee a vibrancy. To illustrate this with a toy example, one street segment next to an office building with ground floor commerce might have a distinct activity pattern than another segment next to a large shopping mall, or from a third segment where a few popular restaurants and bars are located.

### A comparison between ASTs and POI-based street classification framework

This section explores whether POIs associated with street segments are informative for street activities. Seven types of POIs are included as they potentially contribute to pedestrian visits: Accommodation, Civil and Social Service, Education, Open Space, Restaurant and Bar, Retail, and Sports. POI points are then matched with their nearest street segments. Among Boston’s 18,023 street segments, 2127 segments have matched POIs. We apply a simple hierarchical clustering procedure to find representative POI patterns of the matched segments. The dominant POI type is used to name these clusters. If more than one dominant POI type is present, we combine POI types and name them accordingly. As a result, we find seven POI based street segment categories: Retail, Retail and Restaurant, Accommodation and Retail, Accommodation and Restaurant, Education, Civil and Social Service, and Open Space.

**Figure 10:**
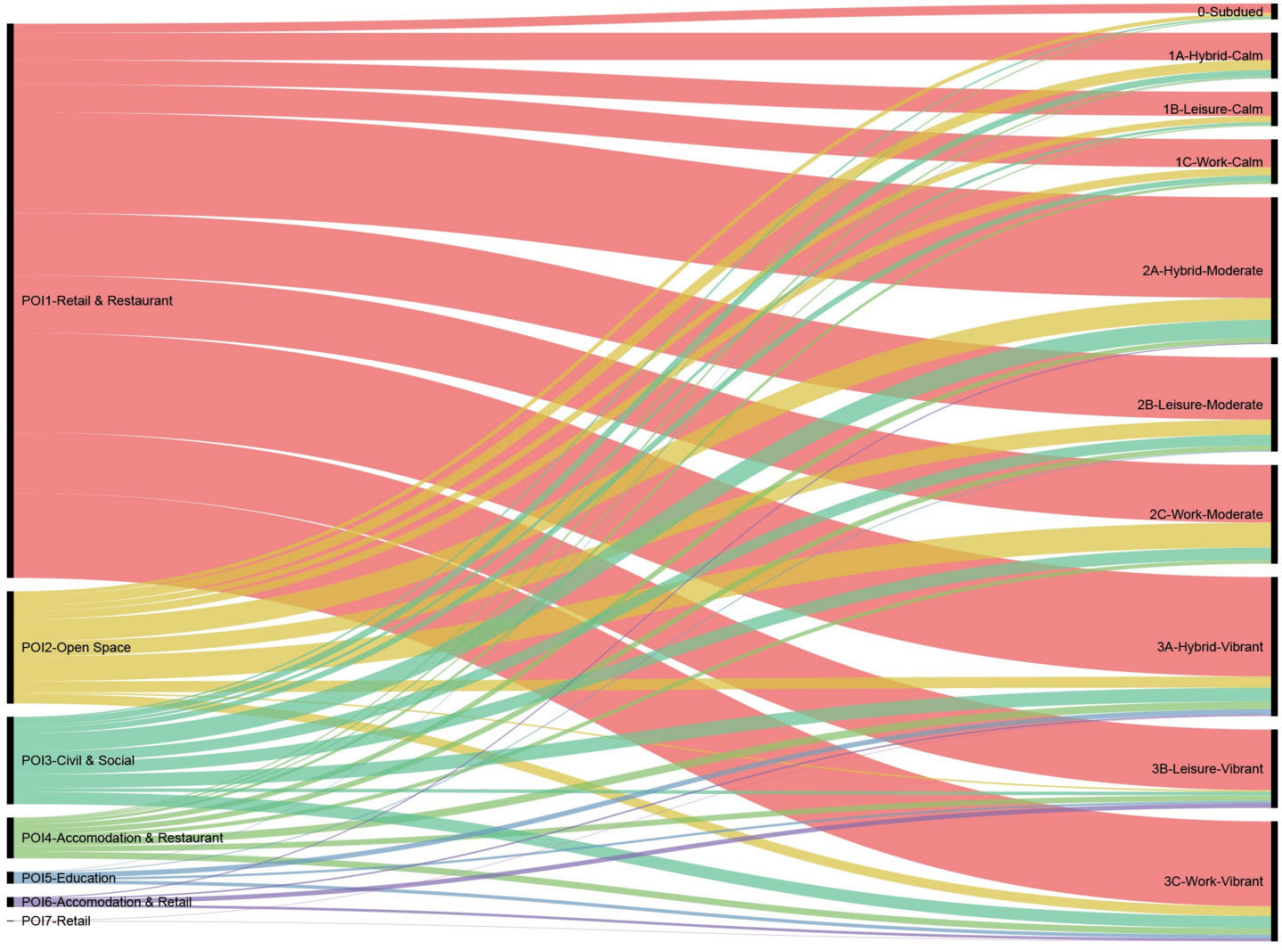
The comparison between POI-based street types (on the left side) and AST results (on the right side)

The small value (0.03) of NMI score indicates limited correlation between POI based category and ASTs. Figure 10 shows the comparison of POI-based segment categories and ASTs for the 2127 street segments. Similar to adjacent land uses, POIs are able to represent some street activities. For example, “Open Space” segments are mainly associated with *Moderate* ASTs. Most of Accommodation and Retail segments belong to *Vibrant* ASTs. However, while most streets are classified into Retail and Restaurant, it occupies a relatively similar proportion in all ASTs. This fact limits the POI-based framework’s predicting power of street activity rhythms, even in this very selected sample.

### Street classification as representation of street activities

As representation of the streets’ dynamics, the ASTs can be related to different urban planning topics that are closely related to human activities. In this section, we show the potential of street classifications as representations of street activities. More specifically, we use regression model as a tool to show the potential of street classification in quantifying the variability of crime across areas.

We obtain crime data from the Boston Police Department,^2^ which includes the time, location and category of reported crimes. To make sure the crime data aligns with our research scope, we selected crimes categories including robbery and assault, which are more related to street activities or happening on streets. The crime dates range from Jan 1, 2018 to Oct 3, 2018. Then, we aggregate these crime incident reports to their nearest street segments (within a 50-meter threshold) for further investigations. As a result, 5835 streets with 18,167 crime is used.

To understand the relationship between street AST and crime, we apply random forest regressors to predict crime count and crime density with our ASTs. By dividing the crime count of each street segment by weekly total activity volumes, we calculate crime count per activity as a proxy for crime density. Table 3 illustrates summary statistics about crime count and density per street segment. Furthermore, we compare the performance of prediction among ASTs, functional street categories and contextual categories. We implement the random forest regressor with following parameters: number of trees is 10, max depth is 3. The prediction is evaluated with Root Mean Square Error (RMSE), which measures the performance on the absolute number.

**Table 3:** Summary of crime count and density per street segment

	Min	Median	Mean	Max
Crime Count	1.0	2.0	3.16	56.0
Crime Density	0.0001	0.048	0.167	17.0

The prediction result is shown in Table 4. We can see that for both crime count and crime density prediction, ASTs achieve better performance over functional and contextual classifications. This shows that our classification framework is able to reveal more information about the streets compared to traditional classifications. The results also point to the potential of using ASTs to understand street dynamics and extending to other applications such as garbage collecting route planning, dynamic curb usage and crowd management.

**Table 4:** Prediction performance (RMSE)

	Crime count	Crime density
AST	4.144	0.481
Functional Category	4.265	0.521
Contextual Category	4.242	0.522

### Limitation

Our study has several limitations. First, the data contains the Christmas period of 2017, which might impact the street activities. We remove the data for December 24, 2017, and re-run the whole analysis to explore such effects. Results show that for Subdued, Moderate, and Vibrant ASTs, more than 90% of the streets are classified as the same type. For Calm streets, about 75% of the street stay the same. It is worth mentioning that the Christmas period also helps our model capture the characteristics of the streets. For example, increasing street activity volumes indicates that such streets are related to holiday activities. In the future work, we will extend the data coverage periods to capture more completed pictures of street activity patterns.

**Figure 11:**
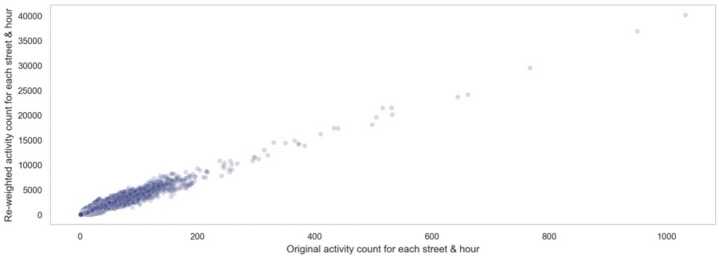
Scatter plot of unweighted and weighted activity count for each street at each hour

Second limitation of our study is that the data involved only covers a sample of the population. Therefore, to test the representatives of our data, we apply a weighting method (post-stratification) [33] which is calculated based on the ratio of recorded users to the true population in the census block group. We calculated the weighted activity count for each street at each hour. Figure 11 shows how activity count changes after post-stratification. Results before and after weighting have a Pearson’s correlation of around 0.96. This shows that our data is able to represent the population to a large extent.

**Figure 12:**
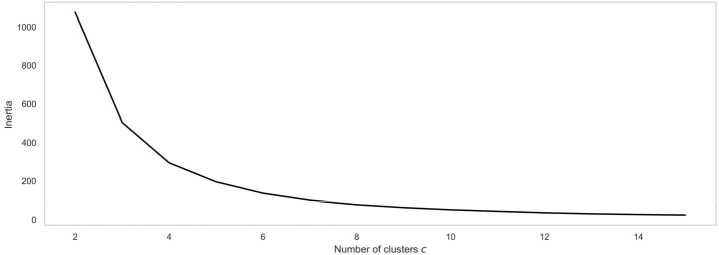
Elbow method to choose best *c* for volume clustering, we choose $c=3$ where the elbow of the curve happens

**Figure 13:**
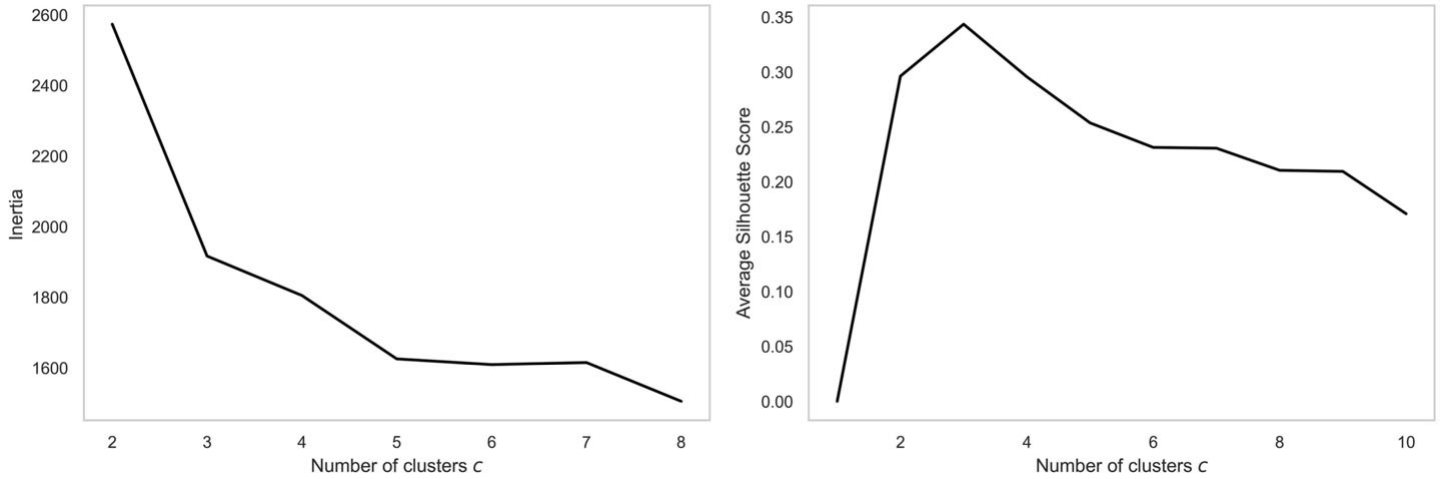
Methods to choose best *c* for pattern clustering, on left plot, elbow of the curve happens at $c=3$ and 5, on right plot, the clustering achieves highest average silhouette score at $c=3$. Combining two plots together, we choose $c=3$ as the best cluster number for pattern clustering

## Conclusion and future work

The contributions of this research are twofold. First, from a methodological perspective, we propose an activity-based street classification framework that emphasizes street segments’ social functions, denoted by street activity rhythms. Inspecting street activities is made possible by using large-scale anonymized and aggregated GPS mobility data. Second, we represent the temporal patterns of street activities as longitudinal activity sequences. Based on the structure of these sequences, we classify street segments with distinct activity volume and pattern characteristics. While many efforts have been made to identify latent patterns in cities, this study is one of the first ones focusing on street or street segment scales, a finer-grained scale.

The empirical contribution of this work emerges from the large-scale application of the framework to Boston’s street segments. In this case study, we identified 10 ASTs street segments with distinct street activity patterns. We find that our proposed approach can highlight street activities that are overlooked in existing frameworks. This finding has important implications for finer-grained urban policymaking and governance, especially for the successful design and management of “Complete Streets.” Although many large cities have advocated the concept of “Complete Street” and proposed new street classification systems to support the street design, most of these systems are incomplete or hard to scale. By applying the proposed framework on street segments, we present an understanding of the sidewalk spaces from the pedestrian perspectives.

Finally, this research also suggests a path for future investigations of street dynamics. We suggest an investigation of street activity patterns of different temporal units. As one of the most dynamic procedures happening in urban spaces, patterns of street activities can evolve across hours, days, weeks, months, seasons, and years. An investigation of the effect of time may be of interest. Meanwhile, we think it would be beneficial to compare ASTs with street classifications extracted from other types of behavior data sets, for example, geo-tagged social media data and urban sensing data. Also, street activities can be sensitive to a broad branch of physical and social factors, such as opening or closing new stores, renovation of urban parks, and election activities. In the context of early 2021, policies and regulations in the context of COVID-19 profoundly changed how people use streets. An in-depth study of these factors and the change of street activity patterns and corresponding ASTs can be valuable.

## Data Availability

The data that support the findings of this study are available from Cuebiq through their Data for Good programme, but restrictions apply to the availability of these data, which were used under license for the current study, and so are not publicly available. Aggregated data used in the models are however available from the authors upon reasonable request and with permission of Cuebiq. Custom code that supports the findings of this study is available from the corresponding author upon request.

## Appendix

The crime data is downloaded from Boston Police Department (BPD).^3^ Each crime record contains latitude, longitude, street name, crime type by offense code, and date. We only consider the outdoor violent crimes happened in 2018. Detailed categories of selected crimes are listed in Table 5.

**Table 5:** Summary statistics for selected crime events in the study area

Offense code group	Offense description	Count
Aggravated Assault	ASSAULT & BATTERY D/W – KNIFE	2
Aggravated Assault	ASSAULT & BATTERY D/W – OTHER	5
Aggravated Assault	ASSAULT & BATTERY D/W – OTHER ON POLICE OFFICER	1
Aggravated Assault	ASSAULT – AGGRAVATED	2561
Aggravated Assault	ASSAULT – AGGRAVATED – BATTERY	4211
Robbery	ROBBERY – STREET	2333
Robbery	ROBBERY – UNARMED – STREET	5
Simple Assault	ASSAULT & BATTERY	3
Simple Assault	ASSAULT – SIMPLE	844
Simple Assault	ASSAULT SIMPLE – BATTERY	13,073

**Table 6:** Street segment selection based on the Census Feature Class Codes (CFCC)

Census Feature Class Codes (CFCC)	Select or not for this research
A15 Primary road with limited access or interstate highway, separated	No
A21 Primary road without limited access, US highways, unseparated	Yes
A25 Primary road without limited access, US highways, separated	Yes
A30 Secondary and connecting road, state highways	Yes
A31 Secondary and connecting road, state highways, unseparated	Yes
A35 Secondary and connecting road, state highways, separated	Yes
A40 Local, neighborhood, and rural road, city street	Yes
A41 Local, neighborhood, and rural road, city street, unseparated	Yes
A45 Local, neighborhood, and rural road, city street, separated	Yes
A60 Special road feature, major category used when the minor category could not be determined	No
A61 Cul-de-sac, the closed end of a road that forms a loop or turn-around	Yes
A62 Traffic circle, the portion of a road or intersection of roads forming a roundabout	No
A63 Access ramp, the portion of a road that forms a cloverleaf or limited-access interchange	No
A64 Service drive, the road or portion of a road that provides access to businesses, facilities, and rest areas along a limited-access highway; this frontage road may intersect other roads and be named	No
A70 Other thoroughfare, major category used when the minor category could not be determined	Yes
A71 Walkway or trail for pedestrians, usually unnamed	Yes
A72 Stairway, stepped road for pedestrians, usually unnamed	Yes
A73 Alley, road for service vehicles, usually unnamed, located at the rear of buildings and property	Yes
A74 Driveway or service road, usually privately owned and unnamed, used as access to residences, trailer parks, and apartment complexes, or as access to logging areas, oil rigs, ranches, farms, and park lands	No

## Footnotes

1 https://data.boston.gov/dataset/boston-street-segments

2 https://data.boston.gov/dataset/crime-incident-reports-august-2015-to-datesource-new-system
